# A timely issue

**DOI:** 10.2478/v10053-008-0066-4

**Published:** 2009-08-03

**Authors:** Marek Binder

**Affiliations:** Psychophysiology Laboratory, Jagiellonian University, Cracow, Poland

**Keywords:** time perception, psychology of time, time estimation, psychophysics, experimental psychology, cognitive psychology

**Figure:**
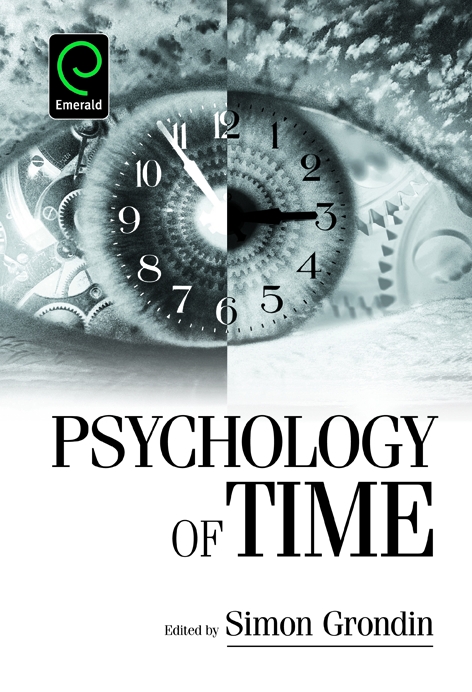
*Psychology of Time* edited by Simon Grondin, Emerald, 2008

The subject of time appears across domains of science and the humanities. One can find it in philosophy, physics, biology, and psychology. In each of these fields, it poses a serious scientific problem and on many occasions leads to considerable debate and even the revision of theories. In psychology, time is equally important both as an objective factor, being a foundation for measuring changes, and as a subjective thing, a target of our perceptions and thoughts. This second aspect of time in psychology is the topic of the anthology edited by Simon Grondin.

This renowned Canadian researcher has undertaken a very ambitious project by extensively exploring a large theme in one volume. As he admits in the Introduction, “The list of impressions we can have about time is long, if not endless, and anyone who takes the time to think about its psychological dimensions remains stunned by this ubiquity, its omnipresence, and its boundless wonder” (p. xi). Luckily, he has succeeded in garnering support from the best researchers in the field.

The book opens with a chapter by Jon E. Roeckelein on the history of the concept of time and the pioneering experimental research on it. The scope of the review spans from antiquity through medieval and modern times, to the research in the middle of 20th century. The chapter traces how the notion of subjective time has departed from the general concept of time throughout the history of human thought. The author reviews many positions and theories, but each of them is described both distinctively and concisely.

The second chapter, by Simon Grondin, focuses on experimental methods in the psychology of time. The author’s intention is to introduce the reader to research methodology as a foundation for understanding the field. The methods are divided into three parts. The first group is called *psychophysical methods* and includes the classical methods for determining the psychophysical function for perceived durations. The next group, termed *animal methods*, covers the methods used in animal research, such as those that examine the influence of reward delay period on conditioning efficacy or those that use operant conditioning for time estimation (temporal differentiation, bisection, and generalization). The final group, *cognitive methods*, relates to the estimation of longer time intervals. Grondin identifies cognitive methods with the first term of Fraisse’s distinction between time estimation and time perception. In the final part of the chapter, the methods for temporal integration and segregation, such as simultaneity judgment and temporal order judgment, are shortly described.

More technical aspects of time perception research are covered in the chapter 3. Hannes Eisler, Anna D. Eisler, and Åke Hellström focus on the issues of psychophysical measurement in the domain of time, such as Vierordt’s law or time-order error. This chapter concludes with the presentation of the parallel-clock model of Hannes Eisler, which is an account of psychophysical function for duration estimation.

In the fourth chapter, Scott W. Brown asks about the relations between temporal processing and attention. He provides ample empirical evidence that attention is a necessary component of temporal processing and that the availability of attentional resources determines the accuracy of temporal judgments.

The next two chapters focus on the auditory modality. In chapter 5, Gertten Hoopen, Ryota Miyauchi, and Yoshitaka Nakajima summarize the main time-based illusions in hearing. They begin with the spatial kappa illusion (when the spatial distribution of sound sources influences perceived temporal intervals between sounds). Among illusions they describe are some they themselves discovered, such as “time shrinking” or “time swelling”. They also present their own theory of creating auditory Gestalts, coined the event construction model. Interestingly, principles of percept formation included in this model are expressed in the form of a series of abstract syntax rules. In chapter 6, Edward W. Large provides accounts of how our brains recognize rhythm in musical patterns and how we adapt our behavior, attention, and perception to it. The author argues that rhythm perception capacity does not reside in some specialized and detached functional module, but that it is probably based on neuronal oscillations, a fundamental feature of the nervous system.

The authors of chapter 7, Howard N. Zelaznik, Rebecca Spencer, and Rich R. Ivry, summarize research on the relations between temporal processing and motor control. They attempt to combine cognitive approaches to human movement timing with accounts based on the dynamical systems theory. The authors reject the notion of a single amodal timing control system, and propose two separate and parallel systems responsible for rhythmic movement: the event and the emergent timing systems. While the former is determined by the salient stimuli from the environment (an example is the finger tapping task), the latter is based on movement control dynamics, which account for the patterns of movements observed in the later stages of circular drawing tasks.

In chapter 8, Trevor Penney and Latha Vaitilingam summarize the contributions of modern neuroimaging methods to the search for neural correlates of temporal processing. One of the main topics of this research is the question of whether there are separate neural mechanisms for subsecond and suprasecond timing. The existence of two separate mechanisms was suggested by the results of behavioral and patient research, and the authors provide an extensive review of neuroimaging studies concerning this issue.

The same problem of separate mechanisms for sub- and suprasecond timing is again reviewed by Thomas H. Rammsayer in chapter 9, this time in the context of neuropharmacological manipulation. The author outlines how pharmacological alteration of neuromodulatory systems can influence time perception. The dopaminergic system is most extensively explored and has generated the biggest body of research, but Rammsayer also describes interesting results for glutaminergic, noradrenergic, and GABA-ergic systems.

Are animals stuck in time? Or, even worse, do they suffer from “temporal myopia”? Perhaps they are bound by “nextism”? All of these curious questions are answered by William A. Roberts in chapter 10, which investigates the sense of time in animals. The author reports some very ingenious experiments aimed at answering the question of whether animals share with humans episodic memory capacity and planning abilities. The question is not straightforward, and the author reports some interesting differences between animal species.

Providing a developmental perspective of time perception is the main topic of chapter 11. William J. Friedman succeeds in summarizing this complicated issue in one chapter. This complexity is also reflected in the different types of developmental changes that pertain to time perception. On one side there are “biologically based temporal abilities” which emerge very early in childhood, and on the other side there are more cognitively based abilities which are predicated on higher cognitive functions like semantic memory, mental imagery, and episodic memory.

The penultimate chapter 12 also deals with longer temporal distances, this time from the subjective side. Richard A. Block and Dan Zakay successfully attempt to present accounts of how we retrieve information about the duration of events in the past, present, and future. The authors describe an intriguing phenomenon of “duration neglect,” in which we have trouble in remembering the duration of painful or happy episodes. For the authors, this is an argument for the conclusion that subjective time does not flow, unless we direct our attention at it (p. 390).

In conclusion, chapter 13, written by the physicist Francesco S. N. Lobo, introduces the reader to how time is understood in physics. The author briefly yet accurately describes the concept of time and causality within the context of the main areas of physics (e.g., special and general relativity, quantum physics). He also presents some unsettled problems, like the question of the objectivity of time passage (block Universe) or its direction (time’s arrow). He also presents some paradoxical solutions to the equations of general relativity, which allow for closed spatiotemporal loops. I think it could be refreshing for a reader of this book, who is likely to come from a psychological background, to juxtapose his knowledge of subjective time with the accounts of the time as an objective entity.

In summary, I am convinced that Simon Grondin has succeeded in preparing a volume that covers the state-of-the-art knowledge about time in psychology. The selection of topics gives potential readers a comprehensive review of various aspects of time processing in different domains of psychology. One thing I miss here is a more detailed account of the problem of the “specious present” and the related issue of continuity and discreteness of perception. I think it is also an important topic in the domain of temporal processing, which is hotly discussed nowadays. Nevertheless, this small gap is thoroughly compensated for by the quality of these authors and the scope of other reviews in this volume. Simon Grondin’s *Psychology of Time* is a necessary purchase for anyone interested in psychology of time.

